# Primary myelolipoma presenting as a nasal cavity polyp: a case report and review of the literature

**DOI:** 10.1186/1752-1947-6-127

**Published:** 2012-05-14

**Authors:** Smiley Annie George, Marie Therese Manipadam, Regi Thomas

**Affiliations:** 1 Department of Histopathology, , Mubarak Al Kabir Hospital, Mubarak Al Kabir Street, Jabriya, Kuwait; 2 Department of Pathology, Christian Medical College, Vellore, Tamil Nadu, India; 3 Department of ENT Surgery, Christian Medical College, Vellore, Tamil Nadu, India

## Abstract

**Introduction:**

Myelolipomas are rare, benign tumors comprising mature adipose tissue and hematopoietic elements. The vast majority occur within the adrenal glands, but extra-adrenal myelolipomas have also been reported in the presacral region, retroperitoneum, mesentery, stomach, spleen, liver, mediastinum and lungs. Here, we present a case of primary myelolipoma occurring in an unusual site: the nasal cavity. To the best of our knowledge, we believe that this location for extra-adrenal myelolipoma has not been previously described in the literature.

**Case presentation:**

We report a case of primary myelolipoma occurring in the nasal cavity of a 48-year-old Asian woman. We describe the etiology, pathology and differential diagnosis of extra-adrenal myelolipomas, and review the literature.

**Conclusions:**

We chose to present this case because of its unusual location. Although myelolipomas are rare, we conclude that they it should be considered in the differential diagnosis of lesions in this site.

## Introduction

Myelolipoma is a rare benign tumor first described in 1905 by Gierke [1] and named by Oberling in 1929. Myelolipomas mostly occur in the adrenal glands, where they are typically non-functioning and asymptomatic. Cases of extra-adrenal myelolipoma are infrequent, with an incidence of 0.4% at autopsy. Only 50 cases have been described in the literature over the last two decades [2]. The occurrence of most extra-adrenal myelolipomas has been noted to be in the presacral region, followed by the retroperitoneum, pelvis, stomach, thorax and peri-renal tissue [2-5].

Myelolipomas are mesenchymal tumors composed of a mixture of fat and bone marrow derived hemopoietic elements. Extra-adrenal myelolipomas occur in women more often than in men and more often in middle-aged to older patients. Typically adrenal and extra-adrenal myelolipomas are asymptomatic, but larger lesions can cause symptoms such as mass effect or hemorrhage [6]. The size of extra-adrenal myelolipoma has been reported to range from 4cm to 15cm, with a mean diameter of 8.2cm. Since myelolipomas are often asymptomatic, their detection via imaging modalities are mostly incidental findings. Malignant degeneration has not been reported so far. Here, we report a case of extra-adrenal myelolipoma in a previously unreported site: the nasal cavity.

## Case presentation

A 48-year-old Asian woman presented to an ear, nose and throat (ENT) surgeon at our facility with complaints of headache and bleeding from the right nasal cavity for 15 days. She was known to be hypertensive and was taking amlodipine and enalapril.

Anterior rhinoscopy revealed a mildly deviated nasal septum and a right nasal polyp. A physical examination did not reveal any other abnormalities. There was no hepatosplenomegaly or lymphadenopathy.

The results of a computed tomography (CT) scan showed a low-density lesion with a few areas of soft tissue density completely filling the right maxillary sinus extending to the middle meatus and posterior nasal cavity, with a widened antrum. There was no bony lesion. The radiological diagnosis was antrochoanal polyp.

**Figure 1:**
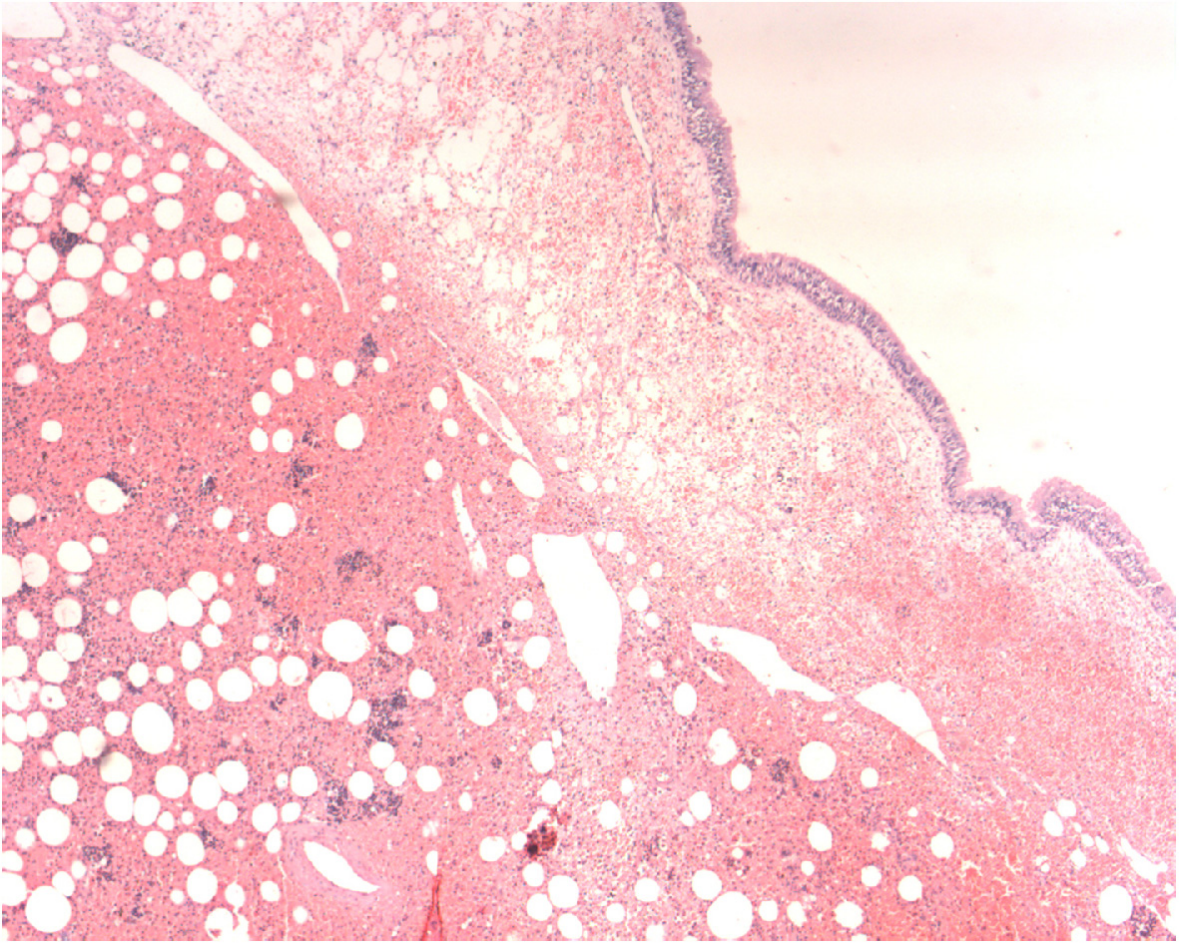
Microscopic picture showing hemopoietic elements mixed with adipocytes beneath the nasal mucosa (hematoxylin and eosin stain, ×100).

**Figure 2:**
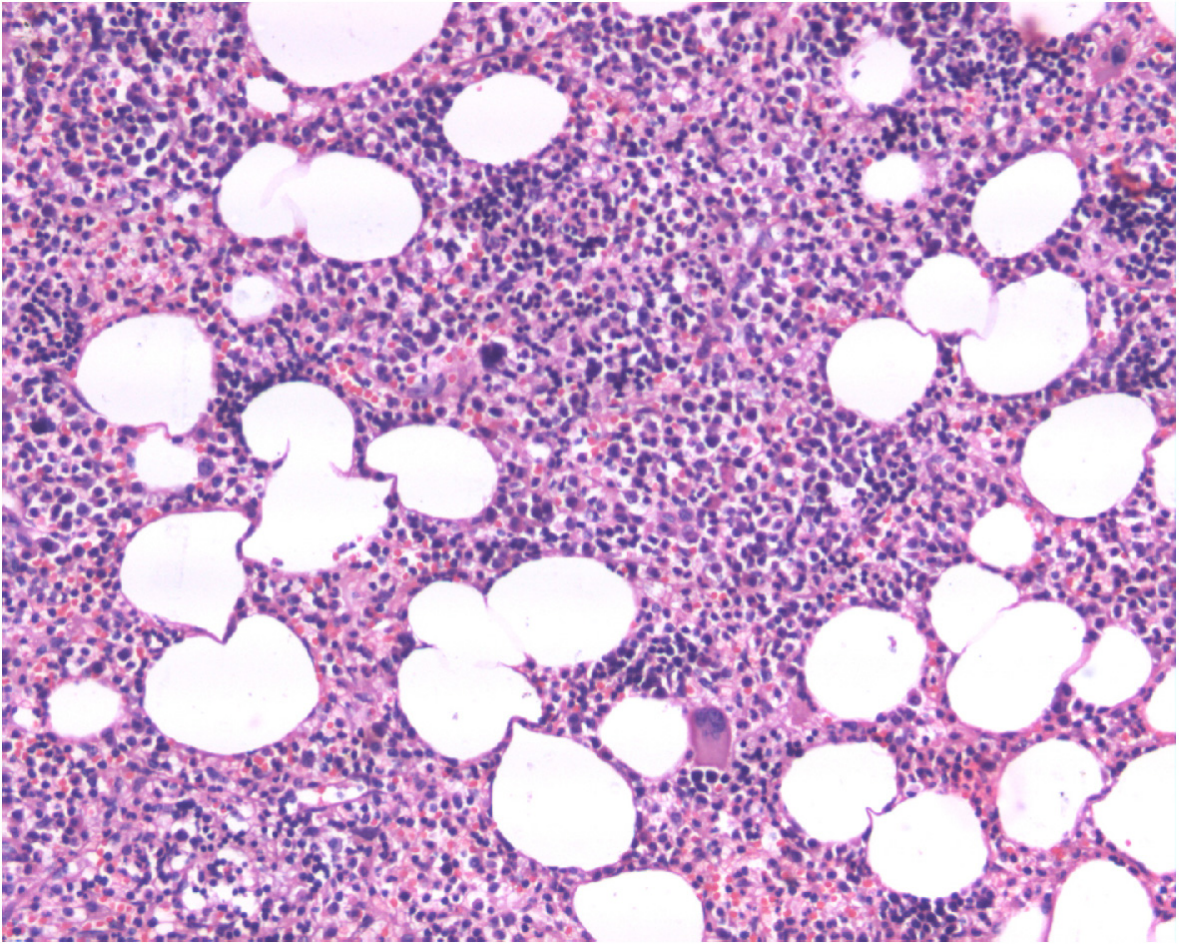
High-power magnification (×200) revealing trilineage hematopoietic elements.

**Figure 3:**
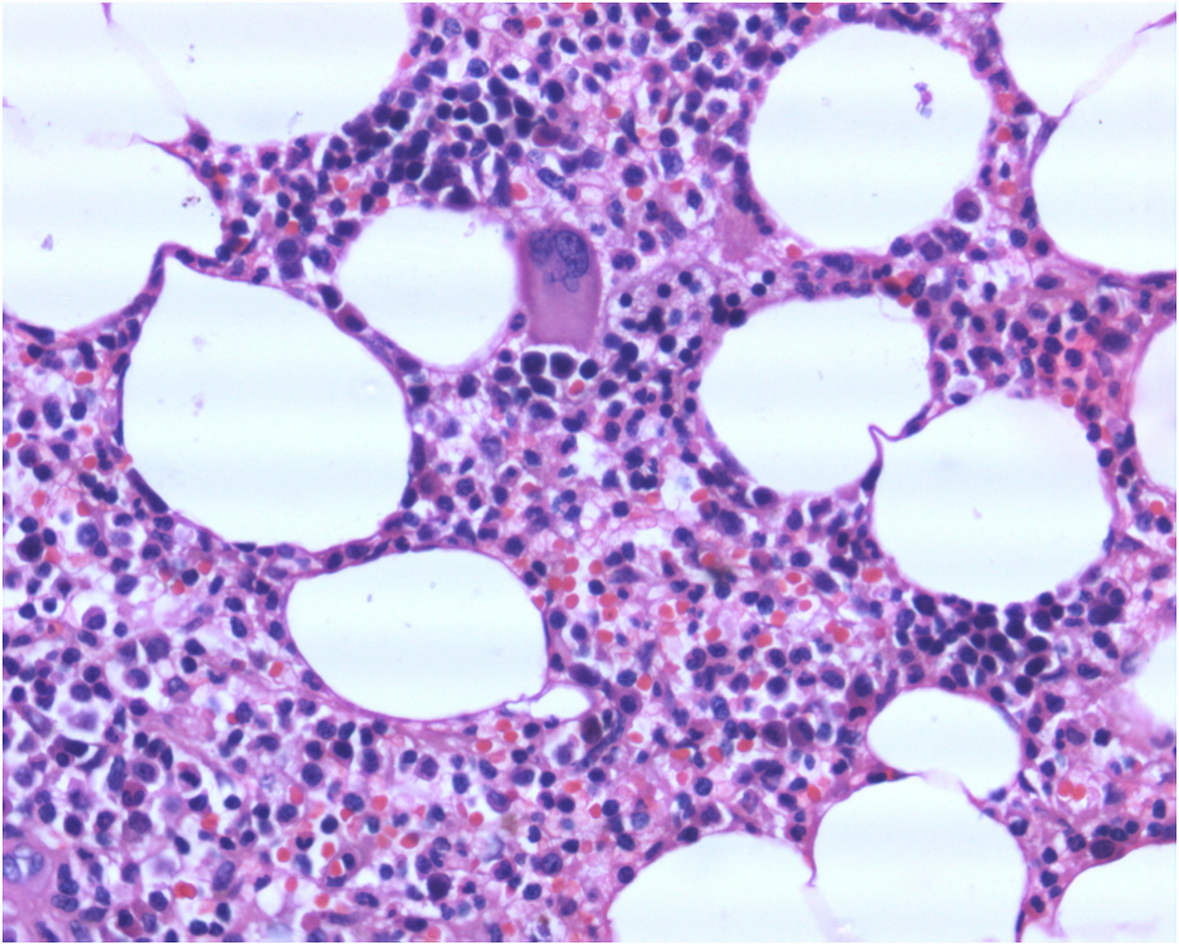
Higher-power magnification (×400) showing normal hematopoietic elements including erythropoietic, granulopoietic and megakaryocytic cell lineages.

The results of all laboratory tests were normal. Subsequently, endoscopic polypectomy and middle meatal antrostomy were performed. Multiple irregular grey to dark brown soft tissue fragments measuring up to 3 × 2 × 1.5cm were sent to our histopathology laboratory. The culture from the surgical specimen was negative for fungus. A histological examination revealed mature adipose tissue mixed with hemopoietic cells (Figure 1). Hemopoietic cells are made up of trilineage elements: myeloid, erythroid and megakaryocytic cells (Figures 2 and 3).

Based on this histomorphology, a hematological investigation including bone marrow aspiration and trephine biopsy were performed to rule out any underlying hematological disorders. Bone marrow aspirate and trephine biopsy were normocellular without fibrosis. Based on these findings, extra-adrenal myelolipoma was diagnosed. Our patient had an uneventful post-operative course and has remained disease-free at 36-month follow-up.

## Discussion

The hypotheses of the cause of the myelolipoma include degenerative changes in hyperplastic tumor cells or adenomas of the adrenal glands, metaplasia in primary stem mesenchymal cells of the adrenal cortex and displacement of differentiated bone marrow cells during embryogenesis [7].

Other authors suggest metaplasia of reticuloendothelial cells caused by prolonged stress, or development of myelolipoma from blood-bone embolic material [8]. Many consider myelolipoma to be a choristoma [9].

Chromosomal translocations (3;21) (q25;p11) detected in myelolipomas and in benign lipomatous neoplasia seen in patients with acute myelogenous leukemia or myelodysplastic syndrome suggest a bone marrow origin of this tumor, and may indicate that myelolipoma is derived from erroneously transferred erythroid cells [10].

Occasionally myelolipomas may be accompanied by endocrine disorders such as Cushing’s disorder, Addison’s disease, Conn’s syndrome, phaechromocytoma, adrenal gland cancer or adenoma, diabetes mellitus or even obesity or hypertension [7].

Therefore, some authors emphasize that myelolipoma may be correlated with prolonged excessive steroid production or genome defects of the endocrine glands responsible for multiple endocrine neoplasia type 1.

Histologically, extra-adrenal myelolipoma should be differentiated from mass forming foci of extra-medullary hematopoiesis. The latter has been previously described in the nasal cavity [11,12].

Mass forming extra-medullary hemopoiesis is symptomatic and is associated with myeloproliferative disorder, hemolytic anemia or severe skeletal disease [13]. Our patient did not have any underlying hematological anormalities.

In contrast to extra-adrenal myelipomas that are well encapsulated, extra-medullary hemopoietic ‘tumors’ lack circumscription and are ill defined.

Microscopically, extra-medullary hemopoietic ‘tumors’ have a predominance of hemopoietic elements with erythroid hyperplasia. Fat is not an enlarged component of the process. Extra-adrenal myelolipomas are composed of a variable proportion of mature adipose tissue and bone marrow cells. The presence of megakaryocytes are considered to be essential for the diagnosis of extra-adrenal myelolipomas [13]. These lesions can have a predominance of the fat component and a more conspicuous lymphocyte population.

Extra-adrenal myelolipoma is distinct from true bone marrow in that no reticular sinusoids or bone spicules are present. However extra-adrenal myelolipomas containing bone spicules have been reported [14]. The bone spicules are thought to be the result of osseous metaplasia.

Another challenge on histomorphology is to differentiate extra-adrenal myelolipomas from lesions that contain hemopoietic tissue and adipocytes, such as teratomas. Histology of the latter shows tissue elements from all the three germ layers.

## Conclusions

We presented this case because of the unusual location of myelolipoma in the nasal cavity. To the best of our knowledge this is the first reported case in the literature of myelolipoma in this location. This case also serves as a reminder that common complaints can be a sign of significant pathology.

We conclude that although myelolipoma is rare, it should be considered in the differential diagnosis of nasal cavity lesions.

## Consent

Written informed consent was obtained from the patient for publication of this case report and any accompanying images. A copy of the written consent is available for review by the Editor-in-Chief of this journal.

## Competing interests

The authors declare that they have no competing interests.

## Authors’ contributions

SG performed the histological examination and was a major contributor in writing the manuscript. MTM supervised the entire case. RT evaluated our patient and performed the surgery. All authors read and approved the final manuscript.
